# Global Assessment of Mycobacterium avium subsp. *hominissuis* Genetic Requirement for Growth and Virulence

**DOI:** 10.1128/mSystems.00402-19

**Published:** 2019-12-10

**Authors:** Marte S. Dragset, Thomas R. Ioerger, Maja Loevenich, Markus Haug, Niruja Sivakumar, Anne Marstad, Pere Joan Cardona, Geir Klinkenberg, Eric J. Rubin, Magnus Steigedal, Trude H. Flo

**Affiliations:** a Centre of Molecular Inflammation Research, Norwegian University of Science and Technology, Trondheim, Norway; b Department of Clinical and Molecular Medicine, Norwegian University of Science and Technology, Trondheim, Norway; c Tuberculosis Research Unit, Germans Trias i Pujol Research Institute, Badalona, Barcelona, Spain; d Department of Immunology and Infectious Diseases, Harvard T. H. Chan School of Public Health, Boston, Massachusetts, USA; e Department of Computer Science and Engineering, Texas A&M University, College Station, Texas, USA; f Department of Infection, St. Olavs University Hospital, Trondheim, Norway; g Department of Biotechnology and Nanomedicine, SINTEF Materials and Chemistry, Trondheim, Norway; Princeton University

**Keywords:** conditionally required genes, *Mycobacterium avium*, *Mycobacterium tuberculosis*, transposon insertion sequencing, virulence genes

## Abstract

Nontuberculous mycobacterial infections caused by the opportunistic pathogen Mycobacterium avium subsp. *hominissuis* (MAH) are currently receiving renewed attention due to increased incidence combined with difficult treatment. Insights into the disease-causing mechanisms of this species have been hampered by difficulties in genetic manipulation of the bacteria. Here, we identified and sequenced a highly transformable, virulent MAH clinical isolate susceptible to high-density transposon mutagenesis, facilitating global gene disruption and subsequent investigation of MAH gene function. By transposon insertion sequencing (TnSeq) of this strain, we defined the MAH genome-wide genetic requirement for virulence and *in vitro* growth and organized ∼3,500 identified transposon mutants for hypothesis-driven research. The majority (96%) of the genes we identified as essential for MAH *in vitro* had a mutual ortholog in the related and highly virulent Mycobacterium tuberculosis (*Mtb*). However, passaging our library through a mouse model of infection revealed a substantial number (54% of total hits) of novel virulence genes. More than 97% of the MAH virulence genes had a mutual ortholog in *Mtb*. Finally, we validated novel genes required for successful MAH infection: one encoding a probable major facilitator superfamily (MFS) transporter and another encoding a hypothetical protein located in the immediate vicinity of six other identified virulence genes. In summary, we provide new, fundamental insights into the underlying genetic requirement of MAH for growth and host infection.

**IMPORTANCE** Pulmonary disease caused by nontuberculous mycobacteria is increasing worldwide. The majority of these infections are caused by the Mycobacterium avium complex (MAC), whereof >90% are due to Mycobacterium avium subsp. *hominissuis* (MAH). Treatment of MAH infections is currently difficult, with a combination of antibiotics given for at least 12 months. To control MAH by improved therapy, prevention, and diagnostics, we need to understand the underlying mechanisms of infection. Here, we provide crucial insights into MAH’s global genetic requirements for growth and infection. We find that the vast majority of genes required for MAH growth and virulence (96% and 97%, respectively) have mutual orthologs in the tuberculosis-causing pathogen M. tuberculosis (*Mtb*). However, we also find growth and virulence genes specific to MAC species. Finally, we validate novel mycobacterial virulence factors that might serve as future drug targets for MAH-specific treatment or translate to broader treatment of related mycobacterial diseases.

## Abstract (precis)

Pulmonary disease caused by nontuberculous mycobacteria is increasing worldwide. The majority of these infections are caused by the Mycobacterium avium complex (MAC), whereof >90% are due to Mycobacterium avium subsp. *hominissuis* (MAH). Treatment of MAH infections is currently difficult, with a combination of antibiotics given for at least 12 months. To control MAH by improved therapy, prevention, and diagnostics, we need to understand the underlying mechanisms of infection. Here, we provide crucial insights into MAH’s global genetic requirements for growth and infection. We find that the vast majority of genes required for MAH growth and virulence (96% and 97%, respectively) have mutual orthologs in the tuberculosis-causing pathogen M. tuberculosis (*Mtb*). However, we also find growth and virulence genes specific to MAC species. Finally, we validate novel mycobacterial virulence factors that might serve as future drug targets for MAH-specific treatment or translate to broader treatment of related mycobacterial diseases.

## INTRODUCTION

Mycobacterium avium complex (MAC) is a group of genetically related and ubiquitously distributed opportunistic mycobacteria that can cause nontuberculous infections collectively called MAC disease (1). M. avium (*Mav*), one of the MAC species, has been classified into subspecies *avium*, *paratuberculosis*, *silvaticum*, and *hominissuis* based on molecular characterizations, prevalent hosts, and diseases caused (2, 3). The latter subspecies, M. avium subsp. *hominissuis* (MAH), can infect humans and lead to pulmonary and disseminated disease, particularly, but not only, in immunocompromised individuals (4). MAH infections are currently hard to treat, with a combination of antibiotics typically given for at least 12 months (5). Similar to its relative M. tuberculosis (*Mtb*), the causative agent of tuberculosis, MAH proliferates within macrophages by hijacking normal phagosomal trafficking, overcoming the host’s elimination strategies (6–12). Mechanisms of infection may therefore partly be conserved between the two species. MAH lacks the type VII ESX-1 secretion system crucial for full *Mtb* virulence (13), suggesting that they also differ in virulence strategies. While *Mtb* is an obligate human pathogen in nature, with limited survival outside the host, *Mav* is environmental and opportunistic, found in a variety of niches (e.g., soil, freshwater, showerheads) and a range of prevalent hosts (3). MAH isolates exhibit high genetic variation (14), perhaps as an adaptation to diverse niches and hosts. It is currently not known to what degree MAH and *Mtb* depend on similar mechanisms for growth and virulence, given the same selective conditions. Even so, MAH genes encoding factors required for basic proliferation and virulence may be attractive targets for improved MAH therapies and may translate to the treatment of related mycobacterial diseases.

**FIG 1:**
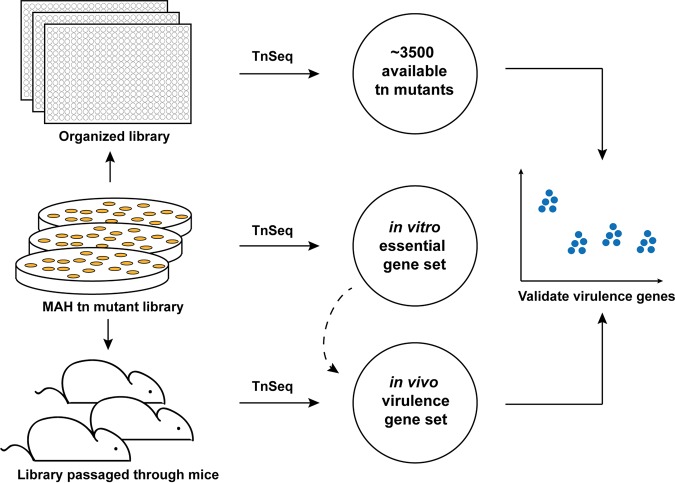
Experimental setup of the study. An M. avium subsp. *hominissuis* (MAH) transposon mutant library of 170,000 mutants (∼66% coverage) was generated by selection on 7H10 agar medium. This library was further subjected to mouse infection or organized mutant by mutant on 384-well plates (24 plates). By TnSeq of the *in vitro*-selected (7H10), *in vivo*-selected (C57BL/6 mice), and the organized library, the MAH essential gene set, and virulence gene sets were defined, and the locations of ∼3,500 organized mutations were identified. The output from the *in vitro*-selected TnSeq library was used to identify virulence genes (dashed line). Finally, a subset of virulence gene hits was validated by mouse infection experiments. Tn, transposon.

Transposon insertion sequencing (TnSeq), which combines transposon mutagenesis and massive parallel sequencing, has been widely used to determine the conditional requirement of bacterial genes on a genome-wide scale (15). By massive parallel sequencing of libraries consisting of more than 100,000 transposon mutants, the genetic requirements of *Mtb*, M. marinum, and M. avium subsp. *paratuberculosis* have been defined for *in vitro* growth or infection (16–20). For research on MAH gene function, transposon mutagenesis has also been of great importance (21–29), although the libraries screened have been limited in size (<5,000 mutants). The first MAH strain with a publicly available, fully assembled genome, was MAH 104 (30). This strain has thus, naturally, been widely studied (e.g., references 8, 10, 11, 30, and 31). MAH 104 is, along with many other *Mav* strains, resistant to transduction by mycobacteriophage TM4 (32), the progenitor of ϕMycoMarT7 (the phage used to generate high-density transposon libraries in other mycobacterial species [16–20, 33, 34]), and transforms with low efficiency (28, 35–37). Therefore, to facilitate genetic engineering and genome-wide investigation of MAH gene function, we identified an MAH strain (MAH 11) highly susceptible to both ϕMycoMarT7-mediated transposon mutagenesis and transformation (another strain, MAH A5, was recently shown to be transducable by ϕMycoMarT7 as well [26, 27]). We used MAH 11 to generate a high-density transposon insertion library (170,000 mutants) with ∼66% saturation density, which we profiled for genes required *in vitro* and in a mouse model of infection. In fact, MAH 11 is currently used as a screening strain in mycobacterial drug discovery programs (38, 39), adding further value to determining the growth requirements of this particular strain. Moreover, we constructed an ordered subset of our transposon library, providing access to ∼3,500 MAH mutants, with which we validated novel MAH virulence factors (the overall experimental setup of our study is summarized in Fig. 1).

## RESULTS

### MAH 11 is susceptible to high-density transposon mutagenesis and transformation.

We aimed to find an MAH strain in which we could create high-density transposon mutant libraries. The transposon donor phagemid ϕMycoMarT7 is widely used (and recommended over Tn*5367* transposition [33]) for efficient mycobacterial transposon mutagenesis (16–20, 34). ϕMycoMarT7 is derived from ϕAE87, which originates from mycobacteriophage TM4 (34, 40). TM4 has been inconsistent in its ability to transduce various *Mav* strains and is unable to infect the commonly studied genome-sequenced strain MAH 104 (32). In agreement with these observations, we failed to obtain kanamycin-resistant mutants (marker for successful transposition of the ϕMycoMarT7-encoded *Himar1* transposon) when attempting to transduce MAH 104. Hence, to identify a ϕMycoMarT7-transducible strain of MAH, we screened seven in-house clinical *Mav* isolates originating from patients at the National Taiwan University Hospital, Taiwan. Around 70% of the strains resulted in various numbers of kanamycin-resistant colonies after transduction, indicating *Himar1* transposition. On the basis of its particularly efficient transducibility (up to 300,000 kanamycin-resistant colonies per ml starting culture in our initial small-scale screen) and general ease to handle, we focused on a strain isolated from an HIV-positive patient’s bone marrow, MAH 11.

**FIG 2:**
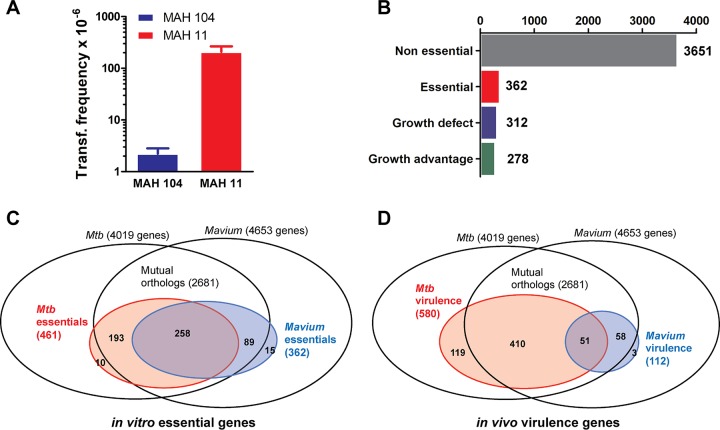
MAH *in vitro* essential and *in vivo* virulence gene sets. (A) MAH 11 and MAH 104 transformation frequencies. Data show means plus standard errors of the means (SEM) (error bars) for three individually electroporated samples. The results are representative of two independent experiments. (B) Number of MAH 11 genes defined as essential, growth defect, growth advantage, and nonessential. (C) Venn diagram illustrating MAH 11 and *Mtb* (as defined by DeJesus et al. [53]) *in vitro* essential genes, relative to the entire pool of MAH 11 (4,653) and *Mtb* (4,019) genes and their mutual orthologs (2,681). MAH 11 (362) and *Mtb* (461) essential genes are shown within the blue and red circles, respectively. A total of 258 genes are essential in both species, 89 (MAH 11) and 193 (*Mtb*) essential genes have a nonrequired mutual ortholog in the other species, and 15 (MAH 11) and 10 (*Mtb*) essential genes do not have mutual orthologs in the other species. (D) Venn diagram illustrating MAH 11 and *Mtb in vivo* virulence (as defined by Zhang et al. [16]) in C57BL/6 mice. MAH 11 (112) and *Mtb* (580) virulence genes are shown within the blue and red circles, respectively. Fifty-one genes cause virulence in both species, 58 (MAH 11) and 410 (*Mtb*) virulence genes have a mutual ortholog in the other species not required for virulence in TnSeq screening, and 3 (MAH 11) and 119 (*Mtb*) virulence genes do not have mutual orthologs in the other species.

*Mav* is notoriously hard to transform (28, 35–37), complicating introduction of new DNA and thus genetic engineering of this species. In our and others’ experience, strain MAH 104 is transformable with low efficiency (36, 41). We investigated the transformation frequency of MAH 11 and found that this strain is around 100 times more susceptible to obtain plasmid DNA compared to the MAH 104 strain, using optimized protocols for *Mav* electroporation (Fig. 2A) (35). In summary, MAH 11 might be particularly apt for high-density transposon mutagenesis and hypothesis-driven genetic approaches.

### MAH 11 genome sequence.

**FIG 3:**
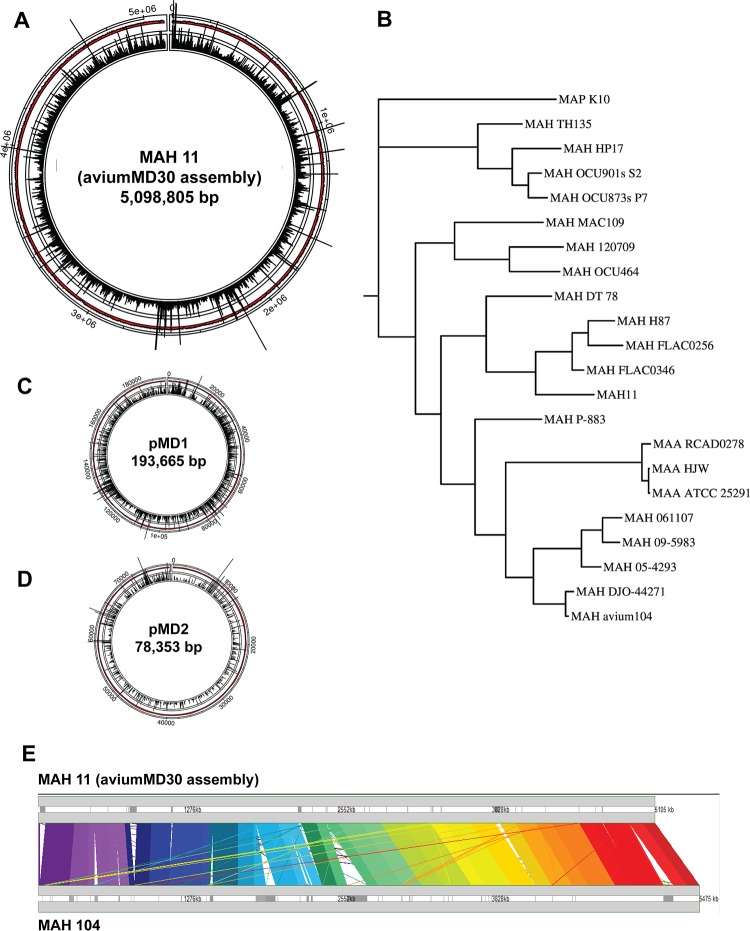
MAH 11 genome and distribution of transposon insertion counts. (A) Transposon insertion counts across the MAH 11 genome. The height of the black bars represents the number of insertion counts at the respective genome site. (B) MAH 11 in a phylogenetic context with 21 other M. avium genomes obtained from NCBI GenBank. (C and D) Transposon insertion counts in pMD1 (C) and pMD2 (D). (E) Comparison of MAH 11 and MAV 104 genomes. Synteny plot showing local sequence homologies between MAH 11 and MAV 104 across the entire lengths of each genome (5.1 Mb and 5.5 Mb, respectively), made with M-GCAT software (77). This plot illustrates that most genes in MAH 11 are present in MAH 104, and the gene order has largely been preserved (i.e., no large-scale chromosomal rearrangements). However, several large regions were deleted (along a few large-scale insertions), and thus, MAH 11 has undergone a substantial reduction of 377 kb in net size.

We sequenced the genome of the ϕMycoMarT7-transducible MAH 11 strain on an Illumina HiSeq 2500 instrument in paired-end mode with a read length of 125 bp, yielding a mean depth of coverage of 55.4. The sequence was assembled by a comparative assembly strategy, using MAH 104 as a reference sequence, augmented with contig building to build large-scale indels. The Illumina data were supplemented with long reads (up to 40 kb) from a PacBio sequencer, which were used to confirm the connectivity of the chromosome. The length of the genome is 5,098,805 bp, and the genome is GC-rich (69.2%) like that of other mycobacteria (Fig. 3A). The genome sequence is highly concordant with the recently reported draft genome of MAH 11 (70 contigs) (42).

MAH 11 is positively identified as M. avium subsp. *hominissuis*, based on 16S rRNA and *hsp65* sequences that are identical to those of MAH 104 (but distinct from other subspecies, like M. avium subsp*. avium*) (2). However, relative to MAH 104, there are substantial numbers of single nucleotide polymorphisms (SNPs) (approximately 10 SNPs per 1 kb) and a cumulative loss of ∼377 kb, showing that it is a distinct lineage (Fig. 3E). The reductions are clustered in several large-scale deletions, listed in Table 1. This variability has been seen in other *Mav* isolates (43, 44), and several of the large-scale deletions correspond to known large-scale polymorphisms (43). In addition, there are several large-scale insertions (Table 1), including a prophage (56 genes, *b6k05_17725*-*18015*, inserted at coordinate 3.77 Mbp), and a cluster of 48 nonphage metabolic genes (*b6k05_03885*-*04170*; 57 kb). The MAH 11 genome contains at least 14 copies of IS*1245* (similar to MAH 104 [45]), but none of IS*901* (associated with members of the MAC complex primarily infecting birds [43, 46]). Figure 3B shows the position of MAH 11 in a phylogenetic tree (created using PHYLIP [http://evolution.genetics.washington.edu/phylip.html]) together with 21 other M. avium genomes obtained from NCBI GenBank, including M. avium subsp. *paratuberculosis* (MAP) K10 and three M. avium subsp. *avium* (MAA) as outgroup strains.

**TABLE 1:** All insertions and deletions of >10 kb in MAH 11 relative to MAH 104^*a*^

Insertion or deletion >10 kb	Coordinate (MAH 11)	Size (bp)	Annotation
Insertions > 10 kb			
B6K05_11270−B6K05_11320	2378550	10,989	Metabolic genes
B6K05_05585−B6K05_05635	1099353	11,986	Metabolic genes
B6K05_10305−B6K05_10440	2149613	29,719	Hypothetical, transporters
BK065_17725−BK065_18015	3779806	41,431	New prophage
BK065_00050−BK065_00255	11154	45,910	Hypothetical, transposases
BK065_03885−BK065_04170	756785	57,435	Metabolic genes

Deletions > 10 kb			
MAV_0683−MAV_0700	639611	10,375	LSP12
MAV_2247−MAV_2267	2068693	14,126	
MAV_2217−MAV_2235	2052758	21,283	LSP10
MAV_1450−MAV_1479	1445064	23,908	LSP14
MAV_0471−MAV_0508	463246	31,473	LSP7
MAV_0253−MAV_0299	293034	40,100	LSP3
MAV_5026−MAV_5107	4888896	81,635	LSP6
MAV_1806−MAV_1974	1800387	162,713	LSP4
MAV_2518−MAV_2691	2378550	173,202	LSP1

a Eight of the >10-kb deletions can be identified with a previous defined list of 14 known LSPs (large-scale polymorphisms) among M. avium strains (43). The remaining six LSPs were present in the MAH 11 genome.

We identified 4,653 open reading frames (ORFs) (along with 1 copy of the rRNAs [16S, 23S, 5S] and 42 tRNAs, similar to MAH 104) using the NCBI Prokaryotic Genome Annotation Pipeline (47). A total of 4,209 genes have mutual orthologs with MAH 104 (where each gene in one organism is the best match for the ortholog in the other organism, with a BLAST E value of <10^−10^; see Data Set S1A in the supplemental material). Almost all of these orthologs (4,123) have ≥96% amino acid identity, and nearly half (1,730) have 100% amino acid identity. For simplicity, we will, where applicable, refer to MAH ORFs using the MAH 104 locus tags (*mav_xxxx*) from here on. Compared to *Mtb* (H37Rv), MAH 11 has ∼500 more genes, and more than half the genes in each genome (2,681) have a mutual ortholog in the other genome (67% of *Mtb* and 58% of MAH total genes) (Fig. 2C and Data Set S1C). Most of the remaining genes also have orthologs, but their E values are above the stringent threshold of 10^−10^ (i.e., 1,052 MAH genes have no clear *Mtb* ortholog [Data Set S1C]), or their specific partner in the other genome is ambiguous, as is sometimes the case in duplicated gene families.

10.1128/mSystems.00402-19.8 DATA SET S1 (A) MAH 104 and MAH 11 mutual orthologs. (B) MAH 11 *in vivo* genetic requirement and MAH 11 and *Mtb* mutual orthologs. (C) MAH 11 and *Mtb* best orthologs. (D) Plasmid pMD1 annotation. (E) Plasmid pMD2 annotation. (F) pMA100 and pMD2 mutual orthologs. (G) MAH 11 *in vitro* genetic requirement. (H) pMD1 *in vitro* genetic requirement. (I) pMD2 *in vitro* genetic requirement. (J) pMD1 (spleen) *in vivo* genetic requirement. (K) pMD1 (liver) *in vivo* genetic requirement. (L) pMD2 (spleen) *in vivo* genetic requirement. (M) pMD2 (liver) *in vivo* genetic requirement. (N) Locations of transposons in MAH 11 organized library. (O) Map of mutants/well in MAH 11 organized library. Download Data Set S1, XLSX file, 1.6 MB. Copyright © 2019 Dragset et al. 2019 Dragset et al. This content is distributed under the terms of the Creative Commons Attribution 4.0 International license.

### MAH 11 plasmids.

Two large extrachromosomal contigs that appear to represent circular plasmids were detected. One, pMD1 (193 kb, 162 ORFs) (Fig. 3C and Data Set S1D), bears weak similarity (based on BLAST search) to parts of plasmids in a wide range of other mycobacteria. The other, pMD2 (78 kb, 66 ORFs) (Fig. 3D and Data Set S1E), is nearly identical to conjugative plasmid pMA100 from MAH strain 88Br (though reduced, since pMA100 is 116 kb [Data Set S1F]) (48) and bears partial similarity to the conjugative pRAW-like plasmids found in several slow-growing mycobacterial species (49).

### MAH *in vitro* essential gene set.

To define genes required for MAH *in vitro* growth, we generated, by ϕMycoMarT7-mediated transduction, a library of ∼170,000 transposon mutants selected on 7H10 medium. The *Himar1*-based mariner transposon of ϕMycoMarT7 inserts randomly at TA dinucleotides (50), of which there are 55,516 sites in the MAH 11 genome (excluding plasmids). We sequenced the transposon junctions of two independent DNA libraries, mapped the genomic positions of the transposon insertion sites (insertion counts), and counted insertions (reduced to unique templates using barcodes [51]) (see Table S1 in the supplemental material). The library had a saturation of 66.3%, with insertions at 36,813 out of 55,516 TA sites. By gene requirement analysis, using a hidden Markov model incorporated into the TRANSIT platform (52), we defined 362 genes as essential for *in vitro* growth, 312 as genes causing growth defects when disrupted, 278 as genes causing growth advantage when disrupted, and 3651 genes as nonessential for growth (Fig. 2B and Data Set S1G). Seventy-one percent (258/362) of MAH 11’s essential genes had an essential ortholog in *Mtb* (as defined by DeJesus et al. [53]). Remarkably, very few MAH (15) and *Mtb* (10) essential genes (∼4% and ∼2% of total essential genes, respectively) did not have a mutual ortholog in the other species (Fig. 2C and Data Set S1G), suggesting that the vast majority of genes required for *in vitro* proliferation are conserved in MAH and *Mtb*.

10.1128/mSystems.00402-19.4 TABLE S1 Output statistics for the sequenced MAH libraries. Download Table S1, PDF file, 0.1 MB. Copyright © 2019 Dragset et al. 2019 Dragset et al. This content is distributed under the terms of the Creative Commons Attribution 4.0 International license.

Almost all genes found on the two MAH 11 plasmids were nonessential for *in vitro* growth (Data Sets S1H and S1I). However, on pMD1, four genes were defined as causing growth defects (though a decrease in insertion counts probably reflects a loss of the plasmid rather than a reduction in growth rate) when disrupted: two genes encoding hypothetical proteins, one homologous to a gene encoding chromosome partitioning protein, *parB*, and one homologous to the DNA processing protein-encoding *dprA*. On pMD2, a gene homologous to *rep*, involved in plasmid replication, caused growth defect when disrupted. Taken together, 674 chromosomal and 5 plasmid MAH genes were defined as essential or causing a growth defect *in vitro* when disrupted.

### MAH 11 establishes infection in mice.

**FIG 4:**
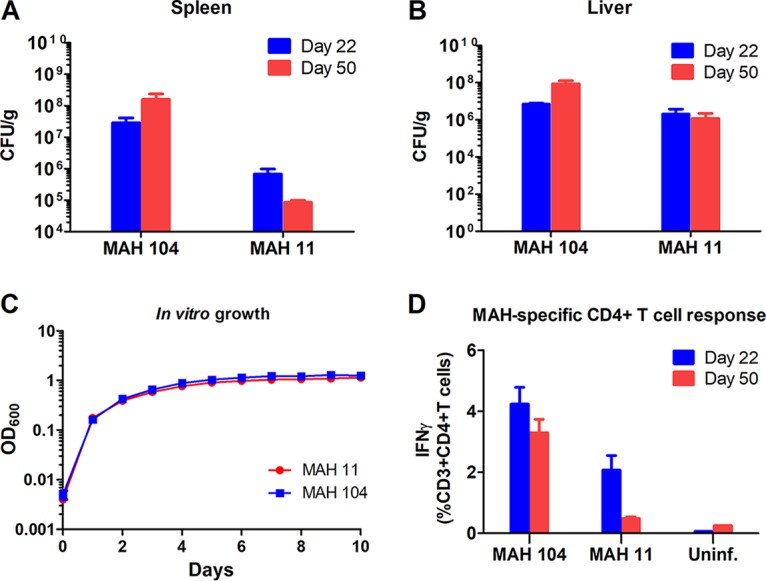
MAH 11 and MAH 104 mouse model infection. (A and B) Bacterial burden in spleen (A) and liver (B) of mice after 22 and 50 days of infection with MAH 104 and MAH 11. Data show means plus SEM for four infected mice in each group. (C) MAH 104 and MAH 11 *in vitro* growth (7H9 medium). Data show means ± SEM for three replicate samples per condition. The results are representative of two independent experiments. (D) Frequencies of MAH-specific CD4^+^ T cells after 22 and 50 days of MAH 104 and MAH 11 infection. Splenocytes from all mice were stimulated overnight with MAH, and frequencies of IFN-γ-producing CD4^+^ effector T cells were analyzed by flow cytometry. Data show means plus SEM for four infected mice per group. Mouse experiments are representative of two independent experiments (for 50 day time point). Uninf., uninfected.

We and others have shown that MAH 104 is virulent in mice (8, 11, 12, 22). We thus examined whether MAH 11 would be suitable to study the role of MAH genes *in vivo*. C57BL/6 mice were infected intraperitoneally with MAH 11 or MAH 104, and organ bacterial load was analyzed in the chronic phase of infection. As we have previously shown, bacterial loads remained relatively constant in liver and spleen from 22 to 50 days after initial MAH 104 infection (12). The same trend was seen for MAH 11, albeit with an overall lower bacterial load compared to that of MAH 104, especially in the spleen (Fig. 4A and B). MAH 11 and MAH 104 grew comparably in 7H9 medium (Fig. 4C), suggesting that MAH 11 is less virulent than MAH 104 in C57BL/6 mice.

T cells produce effector cytokines upon activation to elicit an adaptive immune response toward infections. To control *Mav* infection, production of gamma interferon (IFN-γ) effector cytokine by CD4^+^ T helper 1 cells is of particular importance (54). We have previously monitored antimycobacterial T cell responses to MAH 104 (12). To investigate whether MAH 11 is suitable for studying MAH-specific host immune responses, we measured mycobacterium-specific CD4^+^ T cell responses after mouse infection. Figure 4D shows the frequencies of MAH-specific CD4^+^ T helper 1 cells producing IFN-γ effector cytokine after infection. Interestingly, the frequencies of IFN-γ-producing CD4^+^ T cells were found to be lower in MAH 11-infected mice than in MAH 104-infected mice after 50 days of infection, possibly reflecting the lower organ bacterial loads (Fig. 4A and B).

We performed a broader characterization of the inflammation and tissue pathology in MAH 11-infected mice at day 26 postinfection. In brief, induction of organ homogenate cytokine production was low or not increased in response to infection (tumor necrosis factor alpha [TNF-α] and IFN-γ), except for interleukin 1β (IL-1β), which largely reflected organ bacterial loads (see Fig. S1 in the supplemental material). The overall low induction levels were not surprising; cytokines could be secreted by subsets of immune cells and act in an autocrine/paracrine manner, e.g., in tissue granulomas. As mentioned, we have previously characterized the C57BL/6 infection of strain MAH 104 in great detail (12). Similar to what we observed with MAH 104, MAH 11 infection induced organ pathology seen as disruption of splenic pulp structures, infiltration of immune cells, inflammatory foci (granulomas), and giant cell formation (Fig. S2). In summary, though, MAH 11 appears to be suitable to study mycobacterial disease mechanisms, as well as host responses, in a mouse infection model.

10.1128/mSystems.00402-19.1 FIG S1 Cytokine production during MAH 11 mouse infection. Mice were infected for 26 days with wt MAH 11 or MAH 11 transposon insertion mutants with and without complementation. (A to C) Levels of IL-1β (A), TNF-α (B), and IFN-γ (C) were analyzed in spleen and liver homogenates and serum. Data show means plus SEM of three or four infected mice in each group. The dotted line represents the cytokine level of uninfected mice. Download FIG S1, TIF file, 0.5 MB. Copyright © 2019 Dragset et al. 2019 Dragset et al. This content is distributed under the terms of the Creative Commons Attribution 4.0 International license.

10.1128/mSystems.00402-19.2 FIG S2 Histopathology during MAH 11 mouse infection. Mice were infected with wt MAH 11 or MAH 11 transposon insertion mutants with and without complementation. After 26 days, hematoxylin and eosin staining was performed on spleen and liver sections. The panel shows representative 10× and 40× magnification images of spleen and liver from one out of three or four infected mice in each group. Download FIG S2, JPG file, 2.6 MB. Copyright © 2019 Dragset et al. 2019 Dragset et al. This content is distributed under the terms of the Creative Commons Attribution 4.0 International license.

### MAH virulence gene set.

To identify genes required for MAH virulence, we infected six mice with our MAH library and analyzed bacterial loads from the spleen and liver (organs from two animals each were pooled, resulting in three spleen libraries and three liver libraries) after 26 days of infection. We sequenced the harvested libraries, which yielded saturation of 61.9% and 71.0% (combined over replicates) for spleen and liver, respectively (Table S1). We then defined the genetic requirement for infection using a TRANSIT-incorporated resampling algorithm for comparative analysis (52), comparing output data from sequenced libraries before and after infection. We identified 144 and 128 genes as required for spleen and liver infection, respectively (Data Set S1B). A total of 112 genes were required for survival in both organs (∼80% overlap). Direct comparison of the spleen and liver data sets by resampling did not reveal any statistically significant differences, and hence, no genes uniquely required for colonization of either organ were identified. Among the core genes identified (found in both spleen and liver) were 51 previously identified in *Mtb* mouse model TnSeq experiments (16), including well-established mycobacterial virulence genes like *uvrABC* (the UvrABC endonuclease complex [55]), *secA2* (alternative ATPase of Sec secretion pathway [56]), *icl* (isocitrate lyase [57]), *bioA* (within the biotin biosynthesis pathway [58]), and *glcb* (malate synthase [59]). However, importantly, we identified 61 core genes (92 genes with spleen and liver genes combined) that were not previously detected by *Mtb* TnSeq virulence gene screening (Fig. 2D) (16). Some of these genes were found in genetic clusters, like six genes within the region encoding the type VII ESX-5 secretion system. Other genes were found in operons (for instance, *prcA*/*prcB*, *mav_3300*/*ripA*, and *mav_3691*/*rbfA*) or in close genomic vicinity (*mav_4154*/*4158*/*4159*/*4160/4163*). Strikingly, only three (<3%) core MAH virulence genes did not have a mutual ortholog in *Mtb* (Fig. 2D). Two of these genes, *mav_4273* and *mav_4274*, are potentially coexpressed, and both encode proline-proline-glutamic acid (PPE) family proteins. PPE proteins might show ambiguity in ortholog matching due to large duplications within the family. However, by BLAST search, the two PPE proteins have clear orthologs in MAC species (60), but not in other well-known mycobacterial species like *Mtb*, M. bovis, M. abscessus, or M. leprae (albeit both PPE proteins show a weak similarity to M. marinum PPE14 [*mmar_1235*] with 53 and 57% amino acid identity, respectively). The last of the three MAH virulence genes without *Mtb* mutual orthologs, *mav_4409*, encodes a putative acyltransferase.

As could be expected, most genes found on the two MAH 11 plasmids were nonessential for infection (Data Sets S1J to M); the only exceptions were an AAA-family ATPase on pMD1 (out of 162 ORFs), and two ORFs of unknown function on pMD2 (66 ORFs).

### Identification of mutants in an organized MAH library.

Vandewalle et al. developed a method where sequence tagging transposon library pools was used to bulk identify, by TnSeq, both the gene disrupted and the location of the mutation within an organized (plated) M. bovis BCG transposon library (61). Using a similar approach, we were able to map the specific locations of 2,696 unique transposon insertion mutations within 1,697 (34.8%) of the 4,881 MAH 11 ORFs (plasmid ORFs included) (Data Set S1N). Transposon insertion sites that mapped ambiguously to more than one plate, column, and/or row were disregarded; these might be due to a relatively high number of mutant duplicates in the picked library. A total of 3,161 wells had a unique clone, and only 155 wells had more than one mutant assigned to them (Data Set S1O). The latter might be due to mutants clumping in colonies picked, incomplete sterilization of the robotic picking device between rounds of colony picking, or transposon insertions in repetitive or duplicated regions. To experimentally verify the correct locations of mutations, we sequenced 11 mutants picked from 11 wells (Table S2). All mutants had the transposon inserted at the location predicted by our TnSeq approach. Taken together, we identified the transposon insertion site and mapped the unambiguous location of 3,489 clones (including those in intergenic regions), providing access to a plethora of mutants to study the roles of the respective MAH genes.

10.1128/mSystems.00402-19.5 TABLE S2 Verification of transposon insertion sites by Sanger sequencing. Download Table S2, PDF file, 0.1 MB. Copyright © 2019 Dragset et al. 2019 Dragset et al. This content is distributed under the terms of the Creative Commons Attribution 4.0 International license.

### *uvrB* is required for MAH virulence.

**FIG 5:**
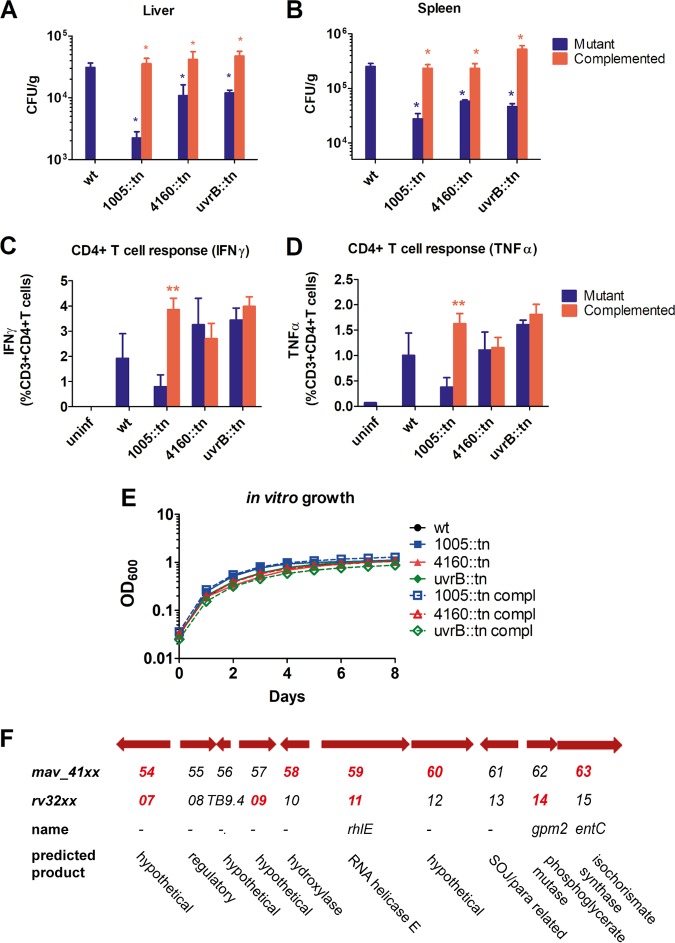
Validation of virulence genes. Mice were infected with wild-type (wt) MAH 11 or MAH 11 transposon insertion mutants with and without complementation. (A and B) After 26 days of infection, the bacterial burden in spleen (A) and liver (B) was determined by CFU per gram organ. Data show means plus SEM of three or four infected mice in each group. Values that are significantly different (*P* ≤ 0.05) by one-tailed Mann Whitney U test are indicated by an asterisk (mutant compared to wt [blue asterisk] or mutant compared to complemented mutant [red asterisk]). (C and D) MAH-specific CD4^+^ effector T cell response in mice infected with wt MAH 11 or MAH 11 transposon insertion mutants. At 26 days postinfection, splenocytes from all mice in each group were stimulated with MAH overnight, and frequencies of IFN-γ-producing (C) and TNF-α-producing (D) CD4^+^ T helper cells were determined by flow cytometry. Data show means plus SEM. Values that are significantly different (*P* ≤ 0.01) from the wt value by two-tailed unpaired Student’s *t* test are indicated by two asterisks. (E) *In vitro* growth (7H9 medium) of wt MAH 11 and transposon insertion mutants with and without complementation. Data show means ± SEM for three replicate samples per condition. The results are representative of three independent experiments. (F) Genetic region spanning from *mav_4154-4163*. Hits from our virulence screen (*mav_41xx*) and previously published *Mtb* virulence screen (Rv32xx) (16) are shown in bold red type.

UvrABC is an enzyme complex involved in Escherichia coli nucleotide excision repair (62). The genes encoding the mycobacterial homologues of the three members of the complex, *uvrA*, *uvrB*, and *uvrC*, were all defined as virulence genes in our screen. *uvrB* has previously been implicated in *Mtb* virulence, via protection against host-mediated reactive nitrogen and oxygen intermediates (55). To verify the involvement of *uvrB* in MAH virulence, we infected mice with an available *uvrB* transposon mutant (*uvrB*::Tn) and complemented mutant for 26 days. The *uvrB*::Tn mutant showed reduced bacterial burden in infected mice compared to mice infected with wild-type (wt) MAH 11 strain and the complemented mutant (Fig. 5A and B). Neither the *uvrB* mutant nor the complemented mutant showed reduced fitness *in vitro* (Fig. 5E); hence, our results suggest that UvrB is required for full virulence in MAH in a mouse model.

### A probable MFS transporter is required for MAH virulence.

Next, we aimed to validate MAH determinants not previously implicated in mycobacterial virulence. A mutant of a probable major facilitator superfamily (MFS) transporter, MAV_1005 (ortholog of Rv0876c), showed attenuated growth in our virulence screen. When we subjected an MAH 11 transposon mutant of this transporter (*1005*::Tn) to mouse model infection, we saw a strong attenuation after 26 days of infection compared to the wt and the complemented mutant (Fig. 5A and B). Furthermore, the mutant, but not the complemented mutant, elicited a reduced MAH-specific CD4^+^ effector T cell response from the host (Fig. 5C and D). The mutant did not show reduced fitness *in vitro* (Fig. 5E). Taken together, our results suggest that *mav_1005* is crucial for MAH virulence in a mouse model.

### A hypothetical gene required for MAH virulence.

Five genes located in close genomic vicinity (region spanning from *mav_4154* to *mav_4163* [Fig. 5F]) appeared as hits in our virulence screen. An insertion mutant of one of the genes, hypothetical gene *mav_4160* (*4160*::Tn), was subjected to a mouse model infection. After 26 days of infection, *4160*::Tn showed attenuated growth in both the liver and spleen (Fig. 5A and B), though the MAH-specific effector CD4^+^ T cell response was not significantly reduced (Fig. 5C and D). The mutant did not show reduced fitness *in vitro* (Fig. 5E). Hence, our findings suggest that *mav_4160* is required for full MAH virulence in a mouse model. No obvious differences in cytokine production or tissue pathology were seen between the mutants tested (*uvrB*::Tn, *1005*::Tn, and *4160*::Tn) and the wt or between the mutants and complemented mutants (Fig. S1 and S2). Nevertheless, the overall organ pathology and IL-1β production grossly reflected bacterial loads, suggesting that these virulence genes might be required for intracellular growth (i.e., adapt to nutrient conditions of the host cells or combat host responses on an intracellular level), rather that affecting organ pathology directly.

## DISCUSSION

Studying the role of genes by loss of function is a powerful approach to understanding how pathogens proliferate and avoid host elimination. We identified a virulent clinical isolate of MAH susceptible to genome-wide high-density gene disruption by ϕMycoMarT7-mediated transposon mutagenesis. The transposon library generated enabled us to define the MAH *in vitro* essential and virulence gene sets using a top-down discovery-based deep-sequencing approach. A total of 674 genes (essential and causing growth defect when disrupted) were identified as required for normal growth *in vitro* (15% of total genes, similar to the proportion of required genes in *Mtb* [53]). There was a substantial overlap (71%) of *in vitro* essential genes between MAH and *Mtb*, as well as many common virulence genes (e.g., *uvrABC*, *secA2*, *icl*) required for survival in a mouse model of infection. The majority of the virulence genes we identified were, however, novel relative to *Mtb* TnSeq virulence screening (16). Likewise, several known *Mtb* virulence factors were not detected in our MAH screen (such as genes involved in cholesterol transport and catabolism, like mammalian cell entry operon [*mce*] 4, *hsaBCD*, and *fadE28-29* [63]), albeit it is important to note that the *Mtb* TnSeq virulence screen identified genes required after 10 and 45 days of infection (as opposed to the 26 days of infection for our MAH screen), potentially causing differences in observed virulence requirement. Surprisingly, though, even with 1,052 genes with no clear *Mtb* ortholog, only 3 of the MAH virulence genes were specific to MAH (i.e., did not have a mutual ortholog in the H37Rv genome). Two of these were PPE family proteins (*mav_4273/4*, found in MAC species but not *Mtb* [60]), which are unique to mycobacteria and associated with virulence, and some have been shown to be secreted by the ESX-5 secretion system in M. marinum and *Mtb* (64, 65). In fact, we observed six genes within the MAH *esx-5* gene cluster among our virulence genes (*mav_2916-2933*), strongly indicating a crucial role of ESX-5 during MAH infection. Interestingly, in *Mtb* and M. marinum, ESX-5 components are required for *in vitro* viability (19, 53), likely as a consequence of their essential role in outer membrane permeability, mediating uptake of nutrients and/or metabolites (66). The same ESX-5 components are dispensable in MAH *in vitro* but required during infection, suggesting that outer membrane permeability might be differently organized in this species. Even so, when we subjected an ESX-5 mutant (*eccA5*::Tn) to mouse infection, we did not see attenuated growth compared to the wt (see Fig. S3A, B and E in the supplemental material), perhaps due to insufficient disruption of gene function in this mutant (transposon insertion in the C terminus) or perhaps this particular gene is not required for ESX-5-mediated virulence in mice, as previously seen for *Mtb* (65). Intriguingly, though, we saw an increased CD4^+^ T cell host response when *eccA5* was overexpressed (Fig. S3C and D). Taken together, it is possible that the two MAC-specific PPE proteins are secreted via the ESX-5 secretion system of MAH. These PPE proteins could be excellent candidates for targeted drug, vaccine, and/or diagnostic discovery for improved control of MAC infections.

10.1128/mSystems.00402-19.3 FIG S3 Mouse infection with MAH 11 *eccA5*::tn ESX-5 mutant. Mice were infected with wt MAH 11 or *eccA5*::Tn mutant with and without complementation (four mice in each group). (A and B) After 26 days of infection, the bacterial burden in spleen (A) and liver (B) was determined by CFU per gram organ. (C and D) MAH-specific CD4+ T cell response in mice infected for 26 days with wt MAH 11 or *eccA5*::Tn mutant with and without complementation. Splenocytes from all mice in each group were stimulated with MAH overnight, and frequencies of CD4^+^ T helper cells producing IFN-γ (C) and TNF-α (D) were determined by flow cytometry. Data show mean plus SEM. *, *P* ≤ 0.05 by unpaired Student’s *t* test (two-tailed) compared to wt. (E) *in vitro* growth (7H9 medium) of wt MAH 11 and *eccA5*::Tn mutant with and without complementation. Data show mean ± SEM for three replicate samples per condition. The results are representative of three independent experiments. Download FIG S3, TIF file, 0.6 MB. Copyright © 2019 Dragset et al. 2019 Dragset et al. This content is distributed under the terms of the Creative Commons Attribution 4.0 International license.

MAH and *Mtb* are both able to persist in human macrophages; however, *Mav* is environmental and opportunistic, while *Mtb* is an obligate human pathogen. The relatively modest overlap between MAH and *Mtb* in mutually orthologous virulence genes (46%) compared to the overlap of *in vitro* essential genes (71%) might reflect different mechanisms of virulence. However, interestingly, many of the novel genes identified in our MAH virulence screen (i.e., not identified in the *Mtb* virulence screening [16]) have been experimentally proven to be required for *Mtb* virulence (exemplified by *prcA* and *prcB* genes encoding proteasome subunits and genes encoding components of the *esx-5* secretion system [65, 67]). This suggests that a portion of the genes we found unique to MAH virulence and that have *Mtb* mutual orthologs might be required for *Mtb* virulence as well. Even so, it is evident that when MAH and *Mtb* are subjected to the same selective conditions (*in vitro* growth on 7H10 agar or *in vivo* growth in C57BL/6 mice), MAH depends on very few genes that do not have mutual orthologs in *Mtb* (∼4% and ∼3%, respectively). Whether this is also true for other MAH isolates remains to be investigated. None of the genes within the major MAH 11 insertions (relative to MAH 104, listed in Table 1) were required for infection, and only four were essential *in vitro*. It has been shown that M. marinum customizes its virulence mechanisms to infect different animal cells (19). It is thus possible that, if subjected to infection of other animal models, a greater proportion of genes specific to MAH would be required.

Interestingly, we identified genes on the two plasmids, pMD1 and pMD2, defined as required for growth *in vitro* or *in vivo*. The *in vitro* growth defect seen in disruption of plasmid replication (*rep*) and partition (*parB*) genes might indicate that the presence of the two plasmids is required for efficient MAH proliferation, that disturbing plasmid replication/partition reduces the global fitness of the MAH cells, or perhaps most likely, that the disruptions cause an underrepresentation of the plasmids due to inefficient replication/partition (but with continued normal growth of the affected bacterial cells). It is currently unclear which role the three plasmid genes we found required *in vivo* might play during infection.

Using defined transposon mutants from our organized (plated) library, we validated a subset of our MAH virulence hits. We verified that the excision repair protein UvrB, a probable MFS transporter, and a hypothetical gene located within a genomic region of several identified virulence genes, are required for full MAH virulence. UvrB has previously been implicated in mycobacterial virulence (55), while the MFS transporter (*mav_1005*) and the hypothetical gene (*mav_4160*) were first validated as mycobacterial virulence factors by us. Of the six other virulence genes identified (based on MAH and *Mtb* screening) in the immediate vicinity of *mav_4160*, two encode hypothetical proteins, one encodes a hydroxylase, one encodes RNA helicase E (RhlE), one encodes isochorismate synthase (EntC), and one encodes phosphoglycerate mutase (Gmp2) (Fig. 5E). EntC was shown to be required for siderophore production in M. smegmatis (68), and might thus play a role in iron acquisition during infection. However, the mechanism of the MFS transporter, as well as *mav_4160* and the surrounding genes, in mycobacterial virulence remains to be elucidated.

*Mav* isolates exhibit high genetic variation (14, 43, 69, 70). In accordance, we registered several large-scale insertions and deletions when we compared the genomic sequence of MAH 11 to the genomic sequence of MAH 104. The movement of genetic material between organisms is mediated by, among other mechanisms, phage transduction, natural transformation, and plasmid conjugation (71). The cause (and effect) of *Mav* genomic plasticity is largely unknown. However, recently, mycobacterial conjugative plasmids have also been identified in *Mav* (48, 49). Interestingly, one of MAH 11’s plasmids, pMD2, is almost identical to previously described *Mav* plasmid pMA100 (48). pMA100 was shown to transfer via conjugation between the slow-growing mycobacteria *Mav* and M. kansasii in a patient with a mixed infection (48). pMD2 might thus partake in genetic exchange between MAH 11 and other mycobacteria.

In conclusion, we identified a highly transformable MAH strain susceptible to ϕMycoMarT7-mediated transposon mutagenesis. This strain enabled genome-wide identification of *in vitro* essential and virulence genes in this species. On the basis of our screens, we identified growth and virulence genes specific to MAH, as well as shared with *Mtb*. MAH-specific genes might be excellent targets for MAC disease control, while shared genes might target related mycobacterial diseases as well. We validated two novel MAH genes required for infection. Since MAH 11 is used as a screening strain in mycobacterial drug discovery programs (38, 39), a comprehensive understanding of the genetic requirement for growth and infection of this strain is a direct asset for current initiatives aimed at discovering new antimycobacterial therapies.

## MATERIALS AND METHODS

### Strains and growth conditions.

Mycobacterium avium subsp. *hominissuis* (MAH) strains used in this study were MAH 104 and MAH 11 (NCBI GenBank accession numbers CP000479 and CP035744, respectively). MAH strains were cultured in Middlebrook 7H9 (BD Difco) supplemented with 0.2% glycerol, 0.05% Tween 80, and 10% albumin-dextrose-catalase (ADC) (50 g bovine serum albumin [BSA] fraction V, 20 g dextrose, 8.5 g NaCl, 0.03 g catalase, distilled water [dH_2_O] up to 1 liter) for liquid growth and in Middlebrook 7H10 (BD Difco) supplemented with 0.5% glycerol and 10% ADC for solid growth. For selection of transposon mutants, 20 μg/ml kanamycin and 0.1% Tween 80 was added to the 7H10 agar plate, the latter to simplify library harvest.

### Genome sequencing and annotation.

DNA from late-log-phase cultures of MAH 11 was extracted using a Masterpure DNA Purification kit (Epicentre), prepared using the TruSeq genome DNA sample preparation kit (Illumina, Inc.), and sequenced on an Illumina HiSeq 2500 instrument in paired-end mode with a read length of 125 bp. The genome sequence was assembled using a comparative assembly method, using MAH 104 as a reference sequence (see Text S1 for a detailed description of the assembly and annotation).

10.1128/mSystems.00402-19.7 TEXT S1 Supplemental Materials and Methods with additional information on genome assembly and annotation, bulk identification of transposon insertion sites in an organized MAH library, and complementation of transposon insertion mutants. Download Text S1, PDF file, 0.4 MB. Copyright © 2019 Dragset et al. 2019 Dragset et al. This content is distributed under the terms of the Creative Commons Attribution 4.0 International license.

### Transformation.

Competent MAH cells were prepared as previously optimized for M. avium (*Mav*) (35). One hundred microliters of competent cells was electroporated with 2 μg plasmid DNA (pMSP12::cfp, kind gift from Christine Cosma and Lalita Ramakrishnan [72]) in a 2-mm cuvette at the following settings: 2.5 kV, 1,000 Ω, and 25 μF. Cells were recovered overnight and plated at serial dilutions for CFU counts. CFU counts of pMSP12::cfp-transformed bacteria selected on 7H10 with 20 μg/ml kanamycin were normalized to the CFU counts of transformed bacteria titered on 7H10 without antibiotics.

### Growth curves.

Wild-type (wt) MAH and mutants were grown in 7H9 medium until they reached stationary phase and then diluted to an optical density at 600 nm (OD_600_) of 0.02 in triplicates of 200 μl 7H9 medium in microplate honeycomb wells (Oy Growth Curves Ab Ltd.). Growth was monitored over the course of 10 days in a Bioscreen growth curve reader (Oy Growth Curves Ab Ltd.) shaking at 37°C.

### Generation of MAH transposon mutant library.

The MAH high-density transposon mutant library was prepared using ϕMycoMarT7 as previously described for M. tuberculosis (*Mtb*) (73), with the exception of growing the bacterial culture to stationary phase as opposed to exponential phase prior to transduction. The amount of ϕMycoMarT7 added for transduction was increased coordinately with the increased bacterial density. Both the phage stock and bacterial culture were heated to 37°C before transduction. The library was incubated at 37°C on 7H10 plates with Tween 80 (0.1%) and kanamycin (20 μg/ml) for 2 to 3 weeks.

### Transposon insertion sequencing.

The transposon library was harvested and pooled by scraping 7H10 plates with 170,000 colonies. Total DNA was purified using Masterpure DNA purification kit (Epicentre) and prepared for transposon insertion sequencing (TnSeq) by PCR amplification of transposon-genome junctions and adapter ligation following the protocol in reference 51. The samples were sequenced on an Illumina GAII instrument, collecting around 10 million 54-bp paired-end reads per sample. The reads were processed using TPP in TRANSIT (52), which counts reads mapping to each TA dinucleotide site (after eliminating reads sharing the same template barcode [51]).

### MAH *in vitro* essential gene set.

Essential genes were identified using a hidden Markov model (HMM) (74), incorporated into TRANSIT (52). The HMM is a Bayesian statistical model that parses the genome into contiguous regions labeled as one of four states—essential (ES), nonessential (NE), growth defect (GD, suppressed insertion counts), or growth advantaged (GA) (insertion counts higher than average)—based on local insertion density and mean read count at TA sites. Thus, the label of each TA site is not determined as the maximum likelihood state independently at each site, but rather, it is the most probable sequence of states for the whole sequence taken together (computed using the Viterbi algorithm), and the call for the gene is based on the majority (or most frequent) call over the TA sites in the gene.

### Mouse infection.

For MAH 104 and MAH 11 infection experiments, groups of four C57BL/6 mice were infected intraperitoneally with 5 × 10^7^ CFU/mouse as previously described (12). On day 22 and day 50 postinfection, MAH-specific effector T cell responses and bacterial load were analyzed. The numbers of CFU per gram of organ were determined by plating serial dilutions of spleen and liver homogenates on 7H10 plates. For the virulence gene screen, six mice were infected intraperitoneally with 6 × 10^7^ CFU/mouse of the MAH 11 transposon mutant library. After 22 days of infection, mice were sacrificed, and the livers and spleens were harvested, homogenized, and plated on 7H10 (livers and spleens from two mice were pooled to make one library, resulting in three liver and three spleen libraries in total from the six mice). After 2 to 3 weeks at 37°C, the colonies were scraped and DNA was prepared for sequencing as described above for TnSeq. For *in vivo* validation experiments of virulence genes, groups of four or five C57BL/6 mice were infected intraperitoneally with (approximately) 7.5 × 10^7^ CFU/mouse MAH 11 wt or MAH 11 transposon insertion mutants. On day 26 postinfection, bacterial load in liver and spleen, MAH-specific effector T cell responses, cytokine levels in organs and serum, and histopathology were analyzed. Statistically significant differences between mutants and the wt were determined using the two-tailed unpaired Student’s *t* test (T cell responses) or one-tailed Mann-Whitney U test (bacterial load by CFU) using Prism GraphPad version 5.

### MAH-specific T cell response.

Isolated splenocytes from infected mice were stimulated overnight with MAH (multiplicity of infection [MOI] of 3:1); protein transport inhibitor cocktail (eBioscience) was added for the last 4 h of incubation. Unstimulated cells were used as controls. Cells were harvested and stained with Fixable Viability Dye eFluor 780 (eBioscience) and fluorescence-labeled monoclonal antibodies against CD3 (fluorescein isothiocyanate [FITC]; eBioscience) and CD4 (Alexa Fluor 700 or Brilliant Violet 605; both from BioLegend). After fixation and permeabilization, intracellular cytokine staining was performed with fluorescent monoclonal antibody against gamma interferon (IFN-γ) (phycoerythrin; eBioscience) and tumor necrosis factor alpha (TNF-α) (allophycocyanin; BioLegend). Cells were analyzed by flow cytometry on a BD LSR II flow cytometer (BD Biosciences), and data were subsequently analyzed with FlowJo (FlowJo, LLC) and GraphPad Prism (GraphPad Software, Inc.) software. Frequencies of IFN-γ- and TNF-α-producing CD4^+^ effector T cells were analyzed from forward scatter (FSC)/side scatter (SSC)-gated, viable CD3^+^ CD4^+^ T cells. The method is described in further detail in reference 12.

### Cytokine measurements.

Interleukin 1β (IL-1β), IFN-γ, and TNF-α levels were analyzed in serum as well as spleen and liver homogenates from infected mice using a custom-made ProcartaPlex immunoassay panel (Affymetrix, eBioscience) according to the manufacturer’s protocol.

### Histopathology.

Standard hematoxylin and eosin staining of spleen and liver sections was performed at the Cellular and Molecular Imaging Core Facility (CMIC) at Norwegian University of Science and Technology (NTNU) as described previously (12). Images were acquired with a Nikon E400 microscope and NIS-Elements BR imaging software (Nikon Instruments, Melville, NY, USA).

### MAH virulence gene set.

For comparative analysis between the *in vitro*-selected and the *in vivo* (mouse infection)-selected transposon libraries, the TRANSIT-incorporated “resampling” algorithm was used (52). Resampling is analogous to a permutation test, examining whether the sum of transposon insertion read counts differs significantly between conditions.

### Bulk identification of transposon insertion sites in the organized MAH library.

A total of 9,216 colonies were picked (384-well format), tagged, and pooled before sequencing on an Illumina HiSeq 2500. The sequences were then subjected to simultaneous detection of location and gene disruption (see detailed description in Text S1 and Table S3 in the supplemental material).

10.1128/mSystems.00402-19.6 TABLE S3 Adapters used in TnSeq of the organized MAH library. Download Table S3, PDF file, 0.1 MB. Copyright © 2019 Dragset et al. 2019 Dragset et al. This content is distributed under the terms of the Creative Commons Attribution 4.0 International license.

### Verification of mutants by Sanger sequencing.

Total DNA was isolated using Masterpure Complete DNA Isolation kit (Epicentre). Sanger sequencing was performed using genomic DNA (gDNA) as the template prepared with BigDye Terminator Cycle Sequencing kit v.1.1 (Thermo Fisher); sequencing was conducted with 60 cycles of PCR, with 1 cycle consisting of 30 s at 95°C, 30 s at 52°C, and 4 min at 60°C, and primer KanSeq2 (CTTCCTCGTGCTTTACGG) reading directly into the gDNA. Samples were purified using BigDye XTerminator kit (Applied Biosystems) and sequenced on an ABI130xl.

### Complementation of transposon insertion mutants.

Plasmids for complementation of transposon insertion mutations were constructed by cloning the wt version of the disrupted gene into the mycobacterium-Escherichia coli shuttle vector pMV261 (75) (swapping the kanamycin resistance gene of pMV261 with the hygromycin resistance gene of pUV15TetORm [76]) for constitutive expression (for more details, see Text S1).

### Ethics statement.

The protocols on animal work were approved by the Norwegian Animal Research Authorities (Forsøksdyrutvalget, FOTS ID 5955). All procedures involving mouse experiments were conducted in accordance with institutional guidelines, national legislation, and the Directive of the European Convention for the protection of vertebrate animals used for scientific purposes (2010/63/EU).
