# Supplementary material for: Global Assessment of Mycobacterium avium subsp. hominissuis Genetic Requirement for Growth and Virulence
Source: mSystems. 2019 Dec 10;4(6):e00402-19. doi: 10.1128/mSystems.00402-19 (PMC6906737; doi:10.1128/mSystems.00402-19)
Supplement: TABLE S1 [file mSystems.00402-19-st001.pdf]

S1 Table. Output statistics for the sequenced MAH libraries.

**Libraries**	**Selective condition**	**Insertion counts (unique mapped templates)**	**Percent TA-sites hit**	**Average count per TA (excluding zeros)**
In vitro 1	7H10 agar	588184	62.4	17
In vitro 2	7H10 agar	553307	61.3	16.3
In vivo S1	Mouse spleen	271603	51.4	9.5
In vivo S2	Mouse spleen	278132	50.0	10.0
In vivo S3	Mouse spleen	151449	46.9	5.8
In vivo L1	Mouse liver	461913	57.3	14.5
In vivo L2	Mouse liver	548881	59.9	16.5
In vivo L3	Mouse liver	2702651	69.6	69.9
