# Supplementary figures and images for: Global Assessment of Mycobacterium avium subsp. hominissuis Genetic Requirement for Growth and Virulence
Source: mSystems. 2019 Dec 10;4(6):e00402-19. doi: 10.1128/mSystems.00402-19 (PMC6906737; doi:10.1128/mSystems.00402-19)

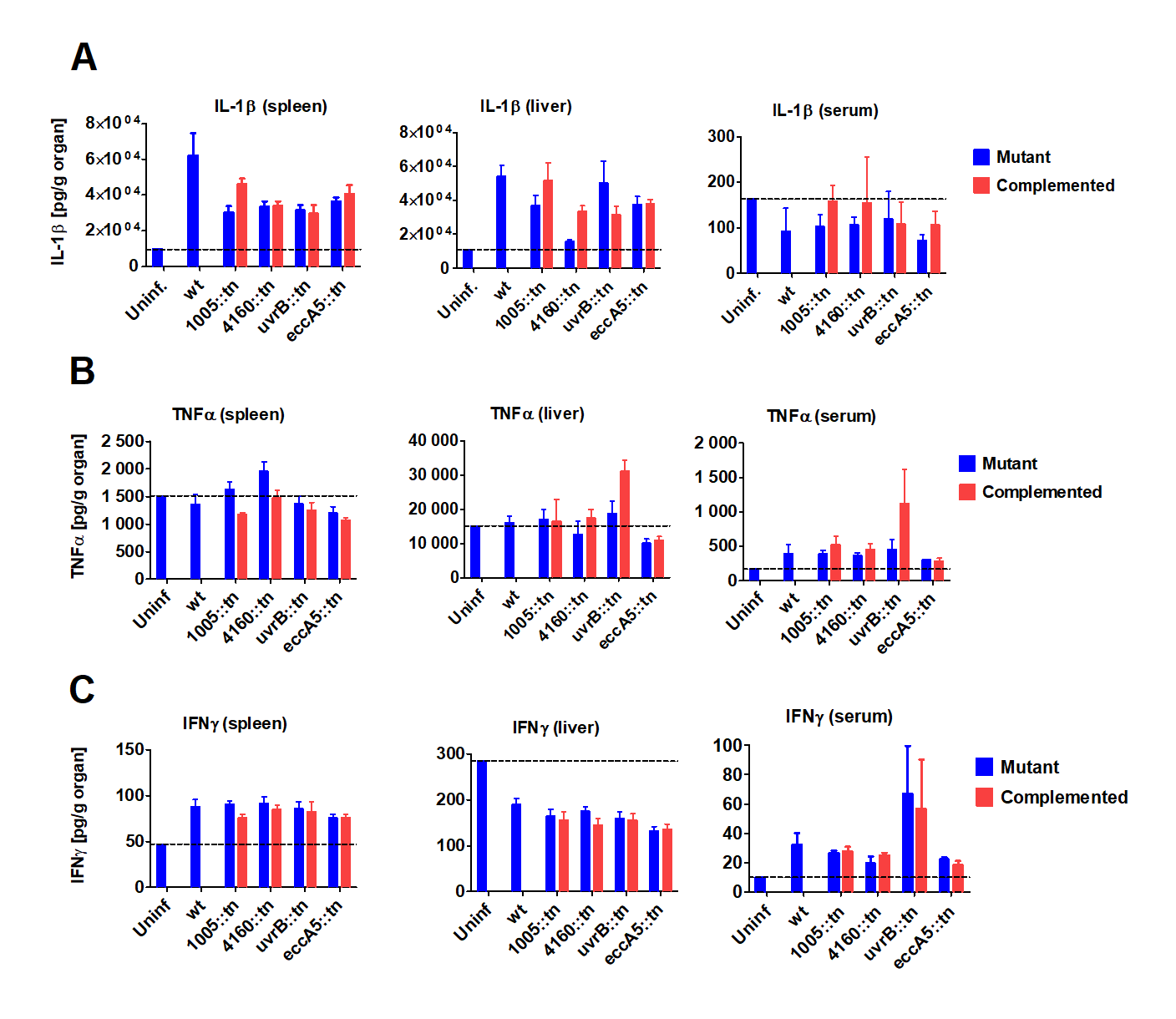

Supplement: FIG S1 [file mSystems.00402-19-sf001.tif]

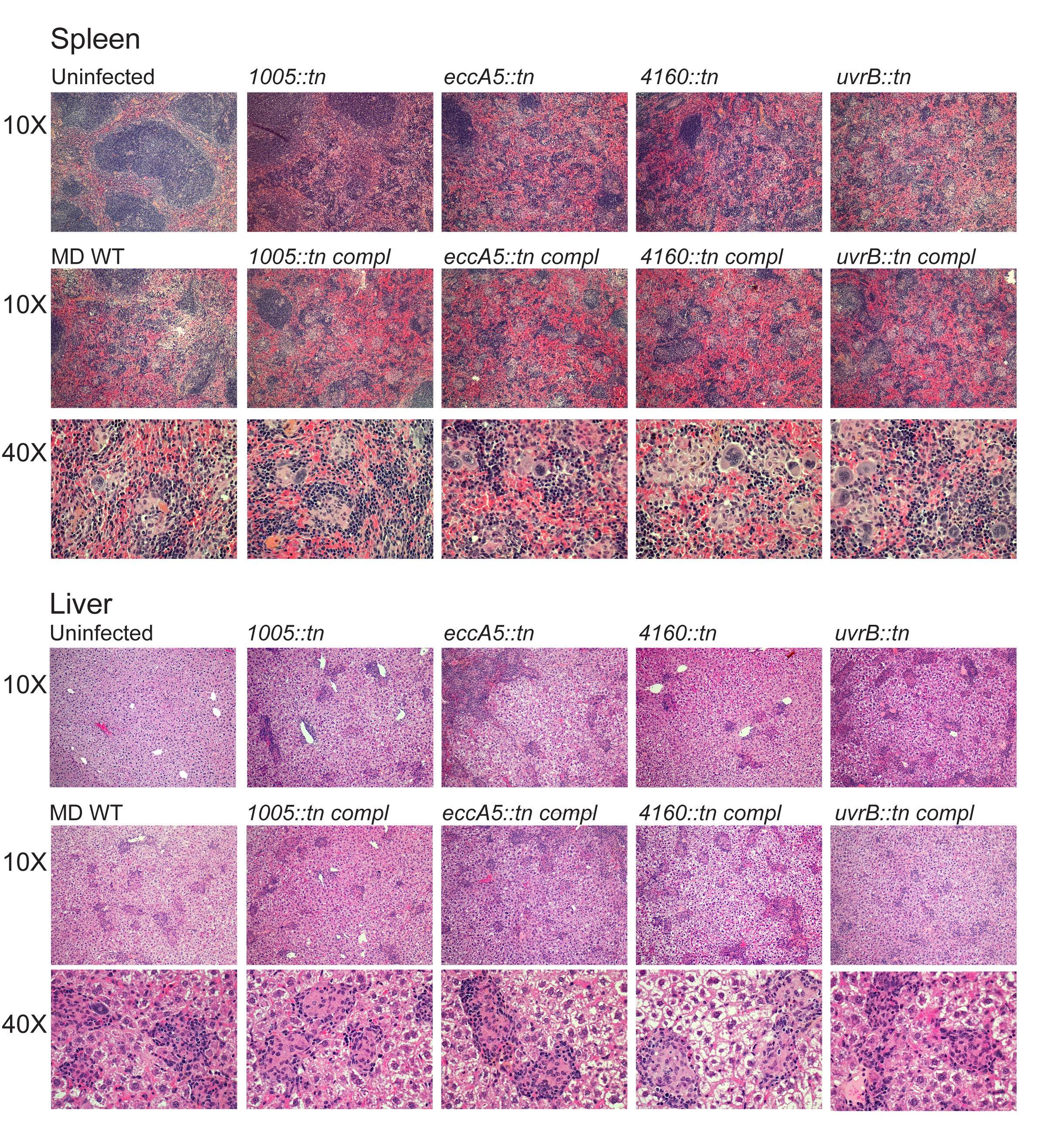

Supplement: FIG S2 [file mSystems.00402-19-sf002.jpg]

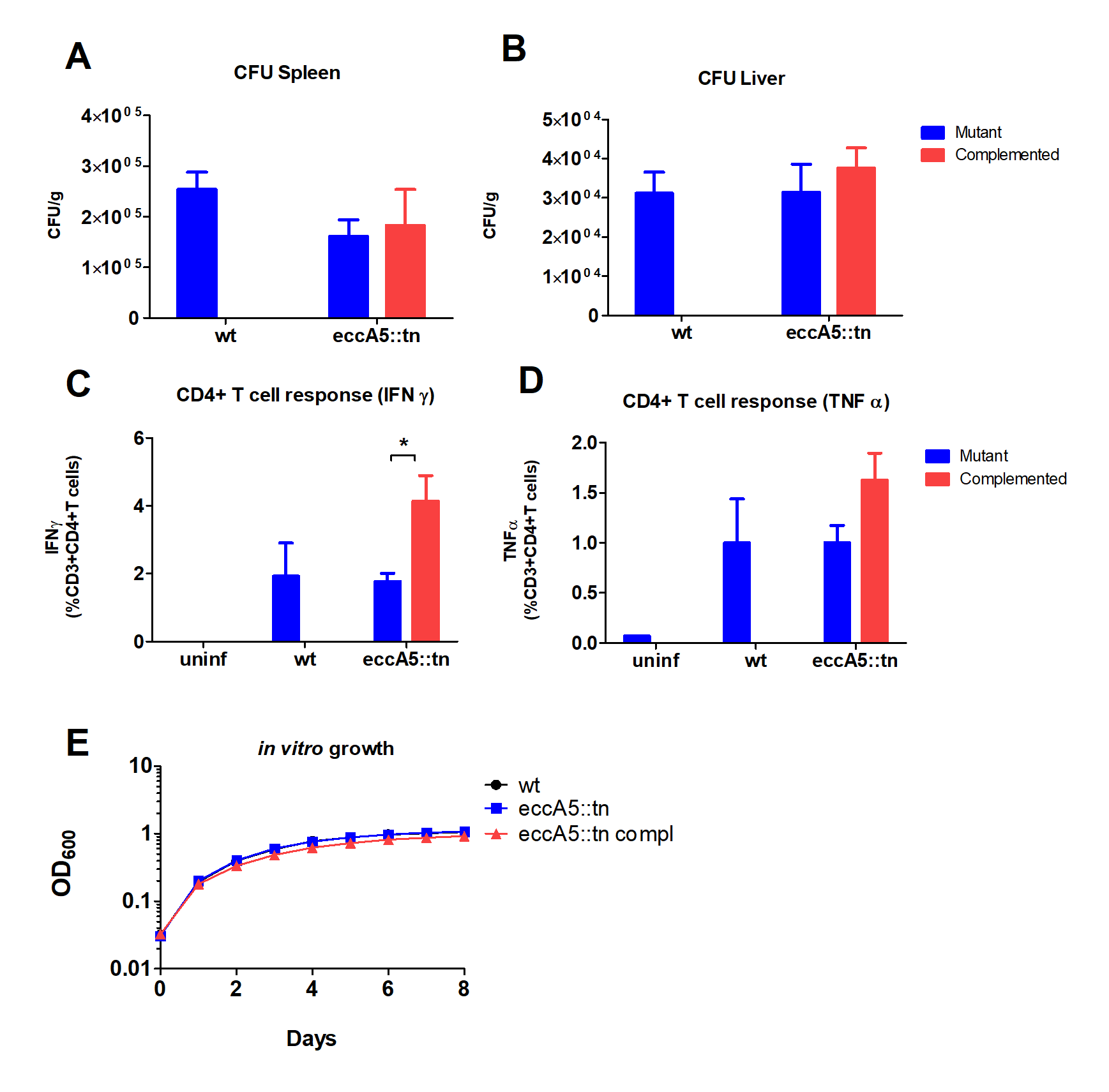

Supplement: FIG S3 [file mSystems.00402-19-sf003.tif]
