# Supplementary material for: Global Assessment of Mycobacterium avium subsp. hominissuis Genetic Requirement for Growth and Virulence
Source: mSystems. 2019 Dec 10;4(6):e00402-19. doi: 10.1128/mSystems.00402-19 (PMC6906737; doi:10.1128/mSystems.00402-19)
Supplement: TABLE S2 [file mSystems.00402-19-st002.pdf]

S2 Table. Verification of transposon insertion sites by Sanger sequencing.

Gene	MAH 104	MAH 11	TA site	Coordinate	Conf.	Sanger match
cpsA	MAV_0673	B6K05_03140	629411	P9 C3 R8	0,877123	yes
cpsA	MAV_0673	B6K05_03140	629732	P16 C22 R9	0,945232	yes
mbtE	MAV_2013	B6K05_08885	1840454	P23 C1 1R13	0,918547	yes
mbtG	MAV_2015	B6K05_08895	1848114	P22 C6 R14	0,892227	yes
mbtN	MAV_2875	B6K05_12225	2565963	P10 C6 R15	0,885609	yes
mbtN	MAV_2875	B6K05_12225	2566020	P21 C8 R16	0,872485	yes
intergenic	MAV_1566	B6K05_07585	1523540	P6 C6 R6	0,856275	yes
mmpL5	MAV_2510	B6K05_11230	2368939	P24 C11 R7	0,879545	yes
mmpL5	MAV_2510	B6K05_11230	2369299	P9 C19 R5	0,943933	yes
mmpL5	MAV_2510	B6K05_11230	2369644	P1 C17 R15	0,931333	yes
mmpL5	MAV_2510	B6K05_11230	2369743	P16 C18 R1	0,954485	yes

Coordinate; refers to plate number (P), column number (C), and row number (R). Conf.; confidence. Sanger match; whether Sanger sequence corresponds with transposon insertion at the predicted TA site (yes) or not.
