# Supplementary material for: Global Assessment of Mycobacterium avium subsp. hominissuis Genetic Requirement for Growth and Virulence
Source: mSystems. 2019 Dec 10;4(6):e00402-19. doi: 10.1128/mSystems.00402-19 (PMC6906737; doi:10.1128/mSystems.00402-19)
Supplement: TEXT S1 [file mSystems.00402-19-s0001.pdf]

S1 Materials and methods.

Genome assembly and annotation. SNPs were identified from mapped reads, which were aligned to the reference sequence using BWA (1) allowing up to 5/125 mismatches, and insertions/deletions (indels) were detected and repaired using local contig-building (as described in (2)). In addition, large-scale insertions and plasmid sequences were assembled de novo using Newbler (Roche, Inc.), and integrated into the genome where connectivity was supported by evidence from paired reads. The genome was annotated using PGAP [48], the NCBI Prokaryotic Genome Annotation Pipeline, which employs GeneMarkS+ to identify ORFs (along with RNAs and pseudo-genes) and assigns putative functions and gene identification based on homology. The sequences have been deposited in NCBI Genbank with accession numbers NBAW00000000 (aviumMD30 assembly of MAH 11), CM009838.1 (pMD1) and CM009839.1 (pMD2). aviumMD30 is the assembly and annotation analyzed in this paper. For genome completion, the MAH 11 strain was re-sequenced on a PacBio Sequal instrument. A total of 650Mb of long reads (up to 40kb) was collected. There were 130,570 subreads, with a mean read length of 4,937. Reads were mapped to the aviumMD30 assembly using blasr (version 5.3.2) (3). Coverage of aligned segments was tabulated at each site. The PacBio data was used to confirm the connectivity of the genome by showing that all sites with low coverage (0-10x) in the Illumina data were spanned by PacBio reads, verifying that the chromosome consists of a single 5.1Mb contig. Similarly, the PacBio reads were aligned to the plasmid sequences pMD1 and pMD2 to confirm their continuity as circular, extra-chromosomal DNA. The updated assembly of the MAH 11 genome sequence (aviumMD36) is deposited in Genbank under accession number CP035744.

Bulk-identification of transposon insertion sites in an organized MAH library.

Organizing the library. A glycerol stock containing the *in vitro*-selected MAH 11 transposon mutant library was plated at a density of 2000 colonies per 23x23 cm 7H10 agar plate with kanamycin and incubated for 2-3 weeks at 37°C. 48 384-well plates were filled with 50 µl 7H9 with kanamycin per well using Biomek NX^P Laboratory Automation Workstation (Beckman Coulter) and software Biomek. 9216 colonies were picked and transferred to 24 384-well plates in duplicate using a Genetix QPixII colony picker and software QSoft XP Picking. The cultures were incubated at 37°C for 3 weeks. To one of the duplicate libraries glycerol was added to each well at a final concentration of 15% using MultiFlo Dispenser (BioTek) and software Liquid Handling Control and stored at -80°C.

Pooling the library. The other duplicate library was pooled by plates (24), columns (24) and rows (16), giving a total of 64 culture pools. Using a Freedom EVO 200 (TECAN) liquid handling robot and software EVOsim, 3 µl from each well position of the 24 plates was pooled together in one master plate (all 24 A1 wells pooled together, all B1 wells pooled together, etc). To ensure enough culture for downstream DNA purification, this was done in triplicate. 24 column pools were created by manually pipetting 30 µl from each well for all 24 columns of the master plate, respectively. The same applied for every row in the master plate, creating 16 row pools. Finally, 10 µl from each well in the original plates was manually pooled to create the 24 plate pools. Of the total 64 pools, each well should be represented three times; in one plate pool, in one column pool and in one row pool.

Sequence-tagging the pools. The various pools were tagged by ligating barcoded adapters to the DNA fragments after DNA purification. Barcodes 1-24 represent plates and columns 1-24, while barcodes 1-16 represent rows 1-16 (S3

Table). Genomic DNA was purified from all 64 pools using Masterpure DNA purification kit (Epicentre), fragmented by Fragmentase (NEB), end repaired by NEBNext End Repair Module (NEB) and A-tailed by NEBNext dA-Tailing Module (NEB). Adapters were constructed by annealing the oligonucleotide Ad1 to Ad2_1-Ad2_24 (containing barcode 1 to 24, S3 Table), and ligated to the respective plate, column and row sample. After adapter ligation, all 24 plate pools, all 24 column pools and all 16 row pools were combined into three master pools, respectively. Transposon junctions within the three master pools were amplified as described previously for TnSeq using a transposon binding and an adapter binding primer, albeit without a nested PCR step (4). Three separate indexes were incorporated into the adapter binding primers for plate, column and row master pools to distinguish them after sequencing.

Analysis of arrayed library of Tn mutants. The three tagged pools were sequenced on an Illumina HiSeq 2500 with 125 bp paired-end reads, collecting 3.2-5.3 million pairs of reads each pool. The genomic portions of the reads (in read 1) were mapped to TA sites in the MAH 11 genome (including plasmids) using BWA (1). The barcodes (8 bp embedded in read 2) were extracted and tabulated for each insertion coordinate. Subsequently, a script was written that compared the TA sites represented by each combination of row-, column-, and plate-barcode to associate each TA site with the most probable well. The count of each barcode for each site was normalized by dividing by the barcode total over all sites. Barcodes with insufficient counts (<10,000 total) were excluded from the calculation. Then the relative frequencies of a plate-, row-, and column-barcodes for each site, $f_{i,p}^{P}$, $f_{i,r}^{R}$, and $f_{i,c}^{C}$ were computed by dividing by the total abundance of plate-, row-, and column barcodes represented by the site. Finally, a score $s(p,r,c) = f_{i,p}^{P} \square f_{i,r}^{R} \square f_{i,c}^{R}$ was

computed for each possible combination of plate, row-, and column-barcodes that represented the likelihood of the well assignment for each TA site *i*. Wells with high probability ($s \geq 0.8$ for the maximal combination) were accepted as unique assignments; wells with $s<0.8$ were rejected as ambiguous (i.e. potentially mapping to multiple wells).

Complementation of transposon insertion mutants. Plasmids for complementation of transposon insertion mutations were constructed by cloning the wt version of the disrupted gene into the mycobacterium-escherichia coli shuttle vector pMV261 (5). To select for the plasmids upon transformation into the mutated strains, the kanamycin resistance gene from pMV261 was swapped with the hygromycin resistance gene of pUV15TetORm (6), creating pMV261H. *b6k05_04950* and *b6k05_04945*, *b6k05_18820*, *b6k05_12440*, and *b6k05_13510* were amplified from the MAH 11 genome and cloned into pMV261H, resulting in plasmids pMV261H *1005*, pMV261H *4160*, pMV261 *eccA5*, and pMV261 *uvrB*, respectively. All clonings were performed using Gibson Assembly® Master Mix (New England Biolabs). Primer sequences can be provided upon request. The complementing plasmids were transformed into their respective MAH 11 mutant, resulting in strains *1005::tn* compl., *4160::tn* compl., *eccA5::tn* compl., and *uvrB::tn* compl.

1. Li H, Durbin R. 2009. Fast and accurate short read alignment with Burrows-Wheeler transform. Bioinformatics 25:1754-1760.
2. Ioerger TR, Feng YC, Ganesula K, Chen XH, Dobos KM, Fortune S, Jacobs WR, Mizrahi V, Parish T, Rubin E, Sassetti C, Sacchettini JC. 2010. Variation among Genome Sequences of H37Rv Strains of Mycobacterium tuberculosis from Multiple Laboratories. J Bacteriol 192:3645-3653.

3. Chaisson MJ, Tesler G. 2012. Mapping single molecule sequencing reads using basic local alignment with successive refinement (BLASR): application and theory. BMC Bioinformatics 13:238.
4. Long JE, DeJesus M, Ward D, Baker RE, Ioerger T, Sassetti CM. 2015. Identifying essential genes in Mycobacterium tuberculosis by global phenotypic profiling. Methods Mol Biol 1279:79-95.
5. Stover CK, de la Cruz VF, Fuerst TR, Burlein JE, Benson LA, Bennett LT, Bansal GP, Young JF, Lee MH, Hatfull GF, Snapper SB, Barletta RG, Jacobs WR, Bloom BR. 1991. New use of BCG for recombinant vaccines. Nature 351:456-60.
6. Ehrt S, Guo XV, Hickey CM, Ryou M, Monteleone M, Riley LW, Schnappinger D. 2005. Controlling gene expression in mycobacteria with anhydrotetracycline and Tet repressor. Nucleic Acids Res 33:e21.
