# Supplementary material for: Global Assessment of Mycobacterium avium subsp. hominissuis Genetic Requirement for Growth and Virulence
Source: mSystems. 2019 Dec 10;4(6):e00402-19. doi: 10.1128/mSystems.00402-19 (PMC6906737; doi:10.1128/mSystems.00402-19)
Supplement: TABLE S3 [file mSystems.00402-19-st003.pdf]

S3 Table. Adapters used in TnSeq of the organized MAH library.

**Adapter**	**Sequence (5' – 3')**
**Ad1**	TACCACGACCA-NH_2
**Ad2_1**	ATGATGGCCGGTGGATTTGTG**ATCACGTT**TGGTCGTGGTAT
**Ad2_2**	ATGATGGCCGGTGGATTTGTG**CGATGTTT**TGGTCGTGGTAT
**Ad2_3**	ATGATGGCCGGTGGATTTGTG**TTAGGCAT**TGGTCGTGGTAT
**Ad2_4**	ATGATGGCCGGTGGATTTGTG**TGACCACT**TGGTCGTGGTAT
**Ad2_5**	ATGATGGCCGGTGGATTTGTG**ACAGTGGT**TGGTCGTGGTAT
**Ad2_6**	ATGATGGCCGGTGGATTTGTG**GCCAATGT**TGGTCGTGGTAT
**Ad2_7**	ATGATGGCCGGTGGATTTGTG**CAGATCTG**TGGTCGTGGTAT
**Ad2_8**	ATGATGGCCGGTGGATTTGTG**ACTTGATG**TGGTCGTGGTAT
**Ad2_9**	ATGATGGCCGGTGGATTTGTG**GATCAGCG**TGGTCGTGGTAT
**Ad2_10**	ATGATGGCCGGTGGATTTGTG**TAGCTTGT**TGGTCGTGGTAT
**Ad2_11**	ATGATGGCCGGTGGATTTGTG**GGCTACAG**TGGTCGTGGTAT
**Ad2_12**	ATGATGGCCGGTGGATTTGTG**CTTGTACT**TGGTCGTGGTAT
**Ad2_13**	ATGATGGCCGGTGGATTTGTG**TGGTTGTT**TGGTCGTGGTAT
**Ad2_14**	ATGATGGCCGGTGGATTTGTG**TCTCGGTT**TGGTCGTGGTAT
**Ad2_15**	ATGATGGCCGGTGGATTTGTG**TAAGCGTT**TGGTCGTGGTAT
**Ad2_16**	ATGATGGCCGGTGGATTTGTG**TCCGTCTT**TGGTCGTGGTAT
**Ad2_17**	ATGATGGCCGGTGGATTTGTG**TGTACCTT**TGGTCGTGGTAT
**Ad2_18**	ATGATGGCCGGTGGATTTGTG**TTCTGTGT**TGGTCGTGGTAT
**Ad2_19**	ATGATGGCCGGTGGATTTGTG**TCTGCTGT**TGGTCGTGGTAT
**Ad2_20**	ATGATGGCCGGTGGATTTGTG**TTGGAGGT**TGGTCGTGGTAT
**Ad2_21**	ATGATGGCCGGTGGATTTGTG**TCGAGCGT**TGGTCGTGGTAT
**Ad2_22**	ATGATGGCCGGTGGATTTGTG**TGATACGT**TGGTCGTGGTAT
**Ad2_23**	ATGATGGCCGGTGGATTTGTG**TGCATAGT**TGGTCGTGGTAT
**Ad2_24**	ATGATGGCCGGTGGATTTGTG**TTGACTCT**TGGTCGTGGTAT

The variable region in each adapter is shown in bold.
